# Applying continuous-time models to ecological momentary assessments: A practical introduction to the method and demonstration with clinical data

**DOI:** 10.1038/s44277-024-00004-x

**Published:** 2024-03-07

**Authors:** Samuel J. Abplanalp, Eric A. Reavis, Thanh P. Le, Michael F. Green

**Affiliations:** 1 Desert Pacific Mental Illness Research, Education and Clinical Center, Veterans Affairs Greater Los Angeles Healthcare System, Los Angeles, CA USA; 2 Department of Psychiatry and Biobehavioral Sciences, University of California, Los Angeles, CA USA; 3 Semel Institute for Neuroscience and Human Behavior, University of California, Los Angeles, CA USA

**Keywords:** Human behaviour, Databases

## Abstract

Ecological momentary assessment (EMA) is a frequently used approach among clinical researchers to collect naturalistic data in real time. EMA data can provide insights into the temporal dynamics of psychological processes. Traditional methods used to analyze EMA data, such as hierarchical linear modeling and multilevel vector auto-regression, paint an incomplete picture of the dynamics of psychological processes because they cannot capture how variables evolve outside predefined measurement occasions. Continuous-time models, an analytical approach that treats variables as dynamical systems that evolve continuously, overcome this limitation. Time advances smoothly in continuous-time models, contrasting with standard discrete-time models in which time progresses in finite jumps. This paper presents a practical introduction to continuous-time models for analyzing EMA data. To illustrate the method and its interpretation, we provide an empirical demonstration of a continuous-time model utilizing EMA data of real-time loneliness and mood states (happiness, sadness, and anxiety) from a clinical sample comprising Veterans with a history of mental illness. Psychological variables, such as feelings of loneliness or sadness, can often change many times throughout the day. However, standard ways of analyzing these variables do not accurately capture these changes and fluctuations. Here, we highlight the benefits of continuous-time models, a method that can capture subtle changes in such psychological variables over time.

## Lay summary

Psychological variables, such as feelings of loneliness or sadness, can often change many times throughout the day. However, standard ways of measuring these variables do not accurately capture these changes and fluctuations. In this paper, we introduce and highlight the benefits of continuous-time models, a method that can capture subtle changes in psychological variables over time.

## Introduction

Ecological momentary assessment (EMA) allows researchers to collect self-report and behavioral data in naturalistic contexts. EMA encompasses a range of methods, including paper and pencil diaries, telephone interactions, and self-monitoring [1]. In recent years, EMA data are almost always derived from smartphones. A typical procedure has participants, via smartphone, complete surveys throughout the day for a specific number of days that ask about real-time thoughts, feelings, or behaviors. Although EMA has the benefit of collecting repeated measurements in real-time within naturalistic environments, traditional methods used to analyze these data paint an incomplete picture of the dynamics of the measures. Specifically, traditional approaches cannot capture how psychological variables evolve, vary, and relate to one another outside predefined measurement occasions [2–6]. Therefore, there is a need to apply methods capable of analyzing these data as dynamical systems that evolve *continuously* over time.

The current paper aims to present continuous-time models for analyzing EMA data. Time advances smoothly in continuous-time models, contrasting with standard discrete-time models in which time progresses in finite jumps [2–6]. Most previous articles on continuous-time models are highly technical and seemingly require readers to have some degree of mathematical background—an assumption that can make such articles daunting to those unfamiliar with the procedures. Hence, we attempt to take a less technical approach (while still introducing general modeling equations) to help clinical researchers understand and interpret continuous-time models from a more practical perspective. First, we briefly describe common discrete-time models, including hierarchical linear modeling (HLM) and the multilevel vector auto-regression (mlVAR) model. Second, we discuss continuous-time models based on first-order stochastic differential equations. Third, we provide an example of a continuous-time model and its interpretation. The example uses EMA data of real-time loneliness and mood states (happiness, sadness, and anxiety) from homeless-experienced Veterans with a history of mental illness.

## Discrete-time models for EMA data

Before introducing methods for analyzing EMA data in discrete time, it is helpful to describe what discrete time means. In discrete time, events are recorded or observed at specific, distinct intervals or time points, and time is treated as a sequence of separate units—not as a continuous flow. For example, a study might measure daily stress levels once per day at 7 p.m. In this case, the time variable is discrete, representing one separable time unit each day. However, the intervals between measurement occasions could be either regular (i.e., occurring at the same time on every occasion) or irregular (e.g., collecting stress data one day at 7 p.m., the next day at 5 p.m. and the following day at 9 p.m.). Sampling at irregular time intervals can allow for a more complete evaluation of the processes under study and has the added benefits of reducing participant anticipation and increasing ecological validity [7]. However, irregular intervals are usually analyzed as regularly occurring intervals— a practice which can bias estimates and lead to inaccurate inferences regarding the temporal dynamics of the variables.

### Hierarchical Linear Modeling

One of the most widely used discrete-time methods for EMA data is hierarchical linear modeling (HLM), also known as multilevel modeling [8]. A foundational feature of HLM is its ability to parse and analyze data into fixed (an effect of a variable that is consistent across all observations) and random (an effect of a variable that varies across observations) effects. In the case of EMA data, repeated measurements are nested within individuals, making it possible for time to be entered into the model as a predictor variable that can capture linear and nonlinear trends across measurement occasions [8]. However, HLM has limitations that hinder its interpretation. First, the modeling of time is restricted. In HLM, time is treated as discrete intervals such that the actual moment when a measurement occurred is not modeled. Instead of treating measurement times as a continuous variable, they are simply modeled as “occasion 1”, “occasion 2”, “occasion 3’” and so forth.

The second limitation of HLM is that time is explicitly entered as a predictor into the model equation, thus treating time as an explanatory variable [2]. Including time assumes that time is the “cause” of other variables. For instance, including time in the HLM of daily stress levels would mathematically model the discrete passage of time as a predictor or cause of later stress. While time may be an explicit cause of certain outcomes (e.g., biological aging), we do not typically assume that time itself causes psychological outcomes in this way. Instead, psychological measures may evolve and interact at every moment as a dynamical system, without time itself as a causal factor. A dynamical system can be broadly described as a set of variables that are treated as a “whole”, and the parts (i.e., variables) that comprise the whole evolve and change over time. One approach that treats variables as a dynamical system is the mlVAR model.

### Multilevel vector auto-regression

The mlVAR model, a multilevel extension of the vector auto-regression (VAR) model, describes dynamic relationships among variables measured repeatedly over time [9, 10]. In mlVAR, lagged regression parameters, or lagged coefficients, are crucial in capturing the temporal dynamics between variables. These coefficients quantify the influence of one variable on itself, called an *auto-regressive effect*, or on another variable, called a *cross-lagged effect*, at a subsequent measurement occasion. Specifically, the lagged coefficients measure the strength and direction of these effects, indicating how a change in one variable can lead to changes in the same or another variable in the future.

Two network structures are estimated from auto-regressive and cross-lagged effects: a lagged network and a contemporaneous network [11]. The lagged network employs lagged coefficients to illustrate the predictive relationships between variables over time, accounting for the influence of all other variables in the model. This network reveals how variables are interconnected through past values and can illustrate temporal precedence and potential causal relationships. The contemporaneous network partials out the lagged coefficients and focuses on the unique relationships among variables within the same time interval. This approach allows for the estimation of immediate, within-time interactions among variables.

A limitation of the mlVAR model is that it relies on the assumption of *equidistance*—an assumption that requires that the difference in time between measurements is equal [11, 12]. Time is only considered implicitly by the order of the measurement occasions, contrasting with HLM, in which time is treated as an explicit predictor. Because time is only considered implicitly, the auto-regressive and cross-lagged results depend on the time interval the researcher chooses. Consequently, separate researchers examining the same variables may get drastically different results depending on the time interval selected. For instance, one study could find a strong auto-regressive effect (i.e., high stability) of stress when measured at 24-hour intervals, and another could observe a weak auto-regressive effect when measured at 1-hour intervals. However, as time is only considered by the ordinal rank of measurement occasions, these effects cannot be compared across studies, making it difficult to understand the true nature of the auto-regressive effect. This problem is referred to as *time-interval dependency*, and it suggests that psychological variables can—and probably often do—evolve and interact outside predefined measurement occasions [5, 13].

Thus, HLM and mlVAR, two common methods of analyzing EMA data, both model time as a discretized variable, but with differing underlying assumptions that are often violated. HLM, despite being able to manage measurements collected at irregular time intervals, treats time as a causal influence that staggers forward in discrete jumps, and does not model variables as dynamical systems. On the other hand, mlVAR treats variables as dynamical systems but suffers from the problem of time-interval dependency. In the next section, we introduce continuous-time models, which overcome these limitations of HLM and mlVAR.

## Continuous-time models for EMA data

Continuous-time models have historically been used in fields such as finance and physics, with a common application being the modeling and prediction of stock prices [14–16]. However, these models have only recently begun to be applied to psychological data [17–19]. The primary objective of continuous-time models is to estimate direct moment-to-moment effects, also known as local dependencies, among variables, which will ultimately allow us to examine the temporal dynamics of variables over *any* time interval.

Using the example of stress, we can assume that measuring stress levels only once per day will not adequately capture its temporal dynamics. Although measuring stress levels every hour would provide richer information, stress likely changes even faster. In fact, there are infinite time intervals between any two discrete measurement occasions [2, 5], meaning that stress would have a value if measured at any imaginable moment. As such, stress and other psychological variables may continuously evolve on a moment-to-moment basis. Such moment-to-moment fluctuations are represented by the “instantaneous rate of change,” which refers to the slope of a variable’s trajectory at any given moment [20]. The slope provides the exact velocity at which a variable increases or decreases, as every point in time has its own rate of change. Unlike discrete-time models, in which changes are assessed between distinct time points, modeling the instantaneous rate of change allows continuous-time models to discern how variables evolve and interact at any conceivable moment in time. In this paper we focus on how continuous-time models can be applied to EMA data, but these models can also be used to analyze other forms of longitudinal data [2].

Moment-to-moment effects from continuous-time models can be calculated using *first-order stochastic differential equations*—mathematical tools used to describe the evolution of systems over time in the presence of both predictable and random influences. The predictable influence of a stochastic differential equation is known as the deterministic trend. For example, the deterministic trend could model how stress is expected to increase during work hours and decrease during relaxation and how this fluctuation in stress affects mood. Given the same starting conditions, it follows a set formula that will always produce the same result. Conversely, the stochastic trend models the randomness or noise inherent in psychological measures that are not explained by the deterministic trend alone. In this paper and in our demonstration, the stochastic trend is represented by a *Weiner process*, which can be thought of as a white noise residual term. Using the stress example, this stochastic trend could be sudden spikes in stress from an unexpected work email that subsequently worsens mood or, conversely, a random act of kindness that significantly improves stress and improves mood. It is also possible for the stochastic trend to model measurement noise inherent within the variables. Of note, while we use first-order stochastic differential equations to estimate continuous-time models in this paper, any differential equation system (e.g., ordinary differential equations, second-order stochastic differential equations) are modeled using a continuous-time framework. We refer interested readers to [21–23] for more technical details related to differential equations.

The concept of equilibrium is essential to interpreting moment-to-moment effects in continuous-time models. A movement away from equilibrium is measured with “drift matrix” parameters that show how variables within the system react over time in response to a random shock—the unexpected changes captured by the model’s stochastic trend. The Weiner process representing the stochastic trend pushes the variables away from equilibrium, and the values of the drift matric parameters determine how the variables respond to the random shocks. When variables are centered and standardized, equilibrium typically corresponds to zero, indicating no deviation from their mean value [5, 24]. The equilibrium state serves as a baseline: any divergence due to a stochastic random shock prompts the system to adjust by moving further from equilibrium or reverting toward it. We can use information about movement toward and away from equilibrium to help interpret *auto-effects* and *cross-effects* within the drift matrix.

**Fig. 1:**
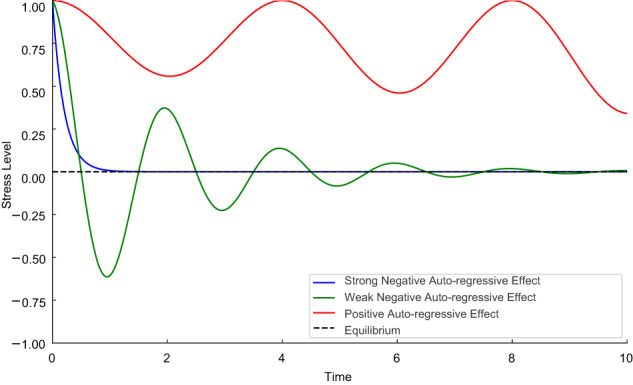
Conceptual illustration of continuous-time model auto-effects. Note. The blue line represents a highly stable, strong negative auto-effect; the green line represents a weak negative auto-effect; the red line represents an unstable positive auto-effect; the dotted black line represents the equilibrium of the hypothetical “Stress” variable.

Auto-effects in continuous-time models differ fundamentally from those in traditional discrete-time models. Traditional auto-regressive effects measure the extent to which a variable at one-time point can predict its future values at later time points. In contrast, continuous-time auto-effects capture the relationship between a variable’s current value and its instantaneous rate of change—again defined as the slope of a variable’s trajectory at any given moment [6, 25]. Specifically, a negative auto-effect indicates a stable effect, suggesting that any increase above equilibrium (a positive deviation) will be followed by a decrease (a return toward equilibrium). Conversely, any decrease below equilibrium (a negative deviation) will be followed by an increase (again, a return toward equilibrium). These dynamics reflect a tendency for the variable to revert to its equilibrium over time. Conversely, a positive auto-effect indicates an unstable effect, as a return toward equilibrium does not necessarily follow increases above or below equilibrium. Figure 1 shows a conceptual illustration of continuous-time model auto-effects using the stress example. The figure includes three types of auto-effects: a strong negative effect, a weak negative effect, and a positive effect. As we can see, the strong negative auto-effect returns to equilibrium very quicky, while the weak negative auto-effect fluctuates above and below equilibrium before returning toward it. Given its instability, the positive auto-effect does not return toward equilibrium.

Cross-effects are conceptually similar to traditional cross-lagged effects in discrete-time models in that they describe the influence of one variable on the future state of another. However, in continuous-time modeling, cross-effects estimate the direct, moment-to-moment relationships between variables [6, 25]. Consider a negative cross-effect from stress to mood. In this scenario, an instantaneous increase in stress corresponds to a decrease in the rate of change at which mood reaches 0. The higher stress levels can impede the recovery of mood, preventing it from stabilizing.

The drift matrix parameters, including auto and cross-effects, are essential values derived from continuous-time models. Once these parameters are calculated, we can use them to predict how variables will evolve and interact continuously over time. Regardless of how frequently the original data were collected, we can use the drift matrix parameters to estimate the relationships between variables over shorter or longer periods [2, 13, 26]. For example, suppose stress and mood data were collected at 1-hour intervals. The drift matrix parameters can be rescaled to explore the dynamics of these relationships on a minute-to-minute basis.

In addition to modeling auto-effects and cross-effects over any time interval, the drift matrix parameters permit us to calculate *impulse response functions* [6, 17]. These functions illustrate how a deviation or random shock (due to the Weiner process stochastic trend) to one variable in the system will affect the evolution of both it and the other variables at subsequent time points. By plotting the trajectory of these responses, we can infer the system’s resilience, identify potential feedback loops, and understand the interdependencies between variables. Impulse response functions may be particularly beneficial in the context of clinical interventions [27]. For instance, if an intervention aims to reduce stress, impulse response functions can help predict how quickly and effectively stress levels might decrease and how this reduction might subsequently affect related variables, such as mood. Moreover, they can inform the duration of an intervention, including whether an intervention is likely to have a short-lived impact or result in sustained changes.

Continuous-time models rely on key assumptions. First, we must assume that there is one underlying continuous-time model (i.e., a single generating process) [2]. In other words, we must think that the variables under study evolve continuously over time. If we believe that the variables do not continuously evolve and instead evolve in discrete intervals (e.g., such as trash collection, which usually happens on a weekly time interval) or event-based intervals (e.g., such as panic attacks only occurring in specific contexts), then a continuous-time model could yield misleading results. A second assumption is that the data used to fit a continuous-time model were collected at time intervals that approximate the genuine dynamics of the variables under study [13]. For instance, if we collected stress data on a year-to-year interval, it would not be appropriate to rescale the drift parameters to time intervals at the minute-to-minute level. Indeed, sampling much slower than a process operates can lead to biased estimates and poor confidence interval coverage, while sampling that approximates or is faster than the process under study results in unbiased estimates [13]. However, in most instances, researchers may not know the genuine dynamics of the variables under study. In these cases, some form of theoretical knowledge about the variables should be used to guide the sampling intervals, as well as the rescaled intervals.

The following section presents an empirical demonstration of a continuous-time model. We utilize EMA data of real-time loneliness and mood states, including happiness, sadness, and anxiety, to illustrate the interpretive value of continuous-time models. The model uses first-order stochastic differential equations to estimate 1) auto-effects that capture the variables’ current value and its instantaneous rate of change across a 24-hour time interval; 2) cross-effects predicting the rate of change of the variables across a 24-hour time interval; and 3) impulse response functions to predict how a random shock to loneliness (and mood states) affects the evolution of both itself and mood states (and loneliness) across a 24-hour time interval.

## Continuous-time model demonstration

Suppose a researcher wishes to use EMA to ascertain the temporal relationships between loneliness and mood states in a clinical sample. Loneliness is a subjective discrepancy between a person’s social needs and the degree to which those needs are satisfied; it is distinct from objective social isolation [28, 29]. According to evolutionary theories, loneliness is a sign that changes in the social environment are not optimal and that the individual should return to social homeostasis [28, 30, 31]. Loneliness should, therefore, be studied and analyzed as a dynamic construct that evolves  continuously over time. Loneliness is distinct from mood states (which may be unrelated to social context); however, the two are frequently associated [32, 33]. Moreover, mood states, like loneliness, may fluctuate throughout the day [34–36]. Thus, using continuous-time models, EMA sampling intervals that measure loneliness and mood states at discrete times throughout the day could be rescaled to examine how these variables evolve on a minute-to-minute basis.

## Materials and Methods

Data were drawn from a study that sought to examine the feasibility of collecting EMA data in homeless-experienced Veterans with a history of mental illness. Participants were recruited from the greater Los Angeles area, had a history of homelessness, and had attained housing with a voucher from the Veterans Affairs (VA) Supportive Housing program in the Department of Housing and Urban Development within the 12 months before study enrollment. Mental illness was broadly defined as participants were eligible if they met DSM-5 criteria for at least one of the following: mood disorder (e.g., major depressive disorder), post-traumatic stress disorder, schizophrenia-spectrum disorder (e.g., schizophrenia), or a substance use disorder (e.g., alcohol use disorder). Diagnoses were confirmed using the SCID-5 Clinician Version [37]. All recruitment and study procedures were approved by the VA Greater Los Angeles Institutional Review Board, and participants provided written informed consent.

The participants were instructed to answer EMA surveys five times a day for seven days using mindLAMP—a smartphone application designed to collect digital phenotyping data (https://docs.lamp.digital). The EMA surveys were sent on a semi-random schedule between 9 am and 9 pm. Participants were scheduled to receive 35 surveys across the seven days and could also manually enter the mindLAMP application and provide additional responses at any time throughout the day. The four EMA items we chose to analyze in this demonstration included: loneliness (“How lonely do you feel right now?”), happiness (“How happy do you feel right now?”), sadness (“How sad do you feel right now?”), and anxiety (“How nervous do you feel right now?”). All items were measured on a 7-point Likert scale, with 0 indicating “not at all” and 7 indicating “extremely.” Participants had to complete at least 10 surveys to be included in the analyses [38].

### Statistical analysis

We applied continuous-time models using the R packages *ctsem* [39] and *ctnet* [5], with a portion of the code adapted from [5, 17]. The R code for all analyses is available at the following OSF page: https://osf.io/jt2k5/. First, we examined sample sociodemographic characteristics and descriptive statistics of the EMA data, including the number of prompts responded to and the time elapsed between prompts.

Second, we used first-order stochastic differential equations to estimate the drift matrix parameters. These parameters reflect the moment-to-moment effects using maximum-likelihood estimation, including auto-effects and cross-effects among loneliness, happiness, sadness, and anxiety. All variables were centered with a mean value of 0. The stochastic differential equations can be expressed by the following:

$$\frac{{{{{{\rm{d}}}}}}{{{{{\boldsymbol{Y}}}}}}(t)}{{{{{{\rm{d}}}}}}t}={{{{{\boldsymbol{AY}}}}}}\left(t\right)+{{{{{\boldsymbol{W}}}}}}(t)$$

Here, $\frac{{{{{{\rm{d}}}}}}{{{{{\boldsymbol{Y}}}}}}(t)}{{{{{{\rm{d}}}}}}t}$ represents the first derivative (i.e., the rate of change) of the variables$\,{{{{{\boldsymbol{Y}}}}}}$ (loneliness and mood states) at time $t$. The derivative is dependent on the momentary values of loneliness and mood states. The drift matrix (${{{{{\boldsymbol{A}}}}}}$) relates the derivative and current values of loneliness and mood states. A Weiner process (a white noise residual term) denoted ${{{{{\boldsymbol{W}}}}}}(t)$ represents the stochastic trend in the equation.

Third, we rescaled the drift matrix parameters to examine auto-effects and cross-effects among the variables over 24 hours (Δ*t* = 0 to Δ*t* = 24). This procedure allowed us to explore how the variables evolve continuously throughout the day. Although rescaling time intervals past 24 hours was mathematically possible, we did not believe rescaling beyond 24 hours would be reliable. Rescaling the drift matrix parameters was done using the following equation:

$${{{{{\boldsymbol{Y}}}}}}\left({t}_{{{\uptau }}}\right)={{{{{{\boldsymbol{e}}}}}}}^{A\Delta {t}_{{{\uptau }}}}{{{{{\boldsymbol{Y}}}}}}\left({t}_{{{\uptau }}-1}\right)+{{{{{\boldsymbol{\epsilon }}}}}}(\varDelta {t}_{{{\uptau }}})$$

Loneliness and mood states at the current measurement occasion, denoted ${{{{{\boldsymbol{Y}}}}}}\left({t}_{{{{{{\rm{\tau }}}}}}}\right)$, are regressed on the values of loneliness and mood states at the previous occasion, expressed by $\left({t}_{{{{{{\rm{\tau }}}}}}-1}\right)$. ${{{{{\rm{\tau }}}}}}$ represents the measurement occasion (e.g., 1, 2, 3, etc.), while $t$ represents the actual time (e.g., 11:30, 13:15, 17:20) that the measurement occurred. These lagged relationships are related via the *matrix exponential* of the drift matrix multiplied by the given time-interval, which is expressed by ${{{{{{\boldsymbol{e}}}}}}}^{A\Delta {t}_{{{{{{\rm{\tau }}}}}}}}$. The vector ${{{{{\boldsymbol{\epsilon }}}}}}\left(\varDelta {t}_{{{{{{\rm{\tau }}}}}}}\right)$ includes the residual terms of loneliness and mood states that are functions of the given time-interval.

Fourth, we calculated impulse response functions. Specifically, we estimated how the variables would evolve and interact over time when loneliness takes on a value of one standard deviation above equilibrium at time 0 (i.e., the current moment in time) or one standard deviation below equilibrium. In addition, we estimated how the variables would evolve and interact over time if the mood state variables (happiness, sadness, and anxiety) took on values one standard deviation above or below equilibrium at time 0. We can calculate the impulse response function by essentially taking the integral form of the equation we used to represent the stochastic differential equation system. This can be expressed using the following:

$${{{{{\boldsymbol{Y}}}}}}\left(t\right)={e}^{{{{{{\boldsymbol{A}}}}}}(t-{t}_{0})}Y\left({t}_{0}\right)+{\int }_{{t}_{0}}^{t}{e}^{{{{{{\boldsymbol{A}}}}}}\left(t-s\right)}d{{{{{\boldsymbol{W}}}}}}(s)$$

${{{{{\boldsymbol{Y}}}}}}\left(t\right)$ represents the state vector of the dynamical system (i.e., loneliness and mood states) at the given time. ${e}^{{{{{{\boldsymbol{A}}}}}}(t-{t}_{0})}$ expresses the matrix exponential of the drift matrix ${{{{{\boldsymbol{A}}}}}}$ multiplied by the time elapsed since the impulse. The term $(t-{t}_{0})$ describes how the system evolves over time from its state at ${t}_{0}$, which is influenced by ${{{{{\boldsymbol{A}}}}}}$. $Y\left({t}_{0}\right)$ represents the state of the system immediately before the impulse, serving as the initial condition for the response function. ${\int }_{{t}_{0}}^{t}{e}^{{{{{{\boldsymbol{A}}}}}}\left(t-s\right)}d{{{{{\boldsymbol{W}}}}}}\left(s\right)$ expresses the integral of the matrix exponential of the drift matrix multiplied by the differential of the Wiener process ${{{{{\boldsymbol{W}}}}}}(s)$ over time from ${t}_{0}$ to $t$. This term captures the effect of stochastic trends on the system’s response to the impulse.

The continuous-time model we estimate here differs from discrete-time models like HLM and mlVAR in important ways. First, this continuous-time model essentially estimates fixed effects, meaning that we assume the effects are consistent across all participants. In a standard HLM, random effects, which are allowed to vary across participants, are also estimated. Although random effects can be included in continuous-time models, we chose to estimate a more simplistic model for illustrative and interpretation purposes. Second, the continuous-time model does not rely on a contemporaneous or lagged network like mlVAR. Contemporaneous and lagged networks would show how loneliness and mood states interact cross-sectionally and over a discrete period (e.g., perhaps averaged across days or via unevenly binned time intervals). In contrast, our continuous-time model shows how loneliness and mood states evolve and interact continuously over an entire day.

## Results

Twenty-five participants were included in the study. Fourteen participants (56%) had a primary diagnosis of major depressive disorder; two participants (8%) had a primary diagnosis of persistent depressive disorder; four participants (16%) had a primary diagnosis of schizophrenia; four participants (16%) had a primary diagnosis of substance use disorder; and one participant (4%) had a primary diagnosis of PTSD. The average age of the sample was 42.33 years old (SD = 12.27); the average education level was 13.00 years (SD = 1.45); 86% of the sample identified as male, and 16% identified as female. The racial breakdown of the sample was as follows: 4% American Indian or Alaskan Native; 60% Black; 8% More than one race; and 28% White.

Regarding descriptive statistics of the EMA data, participants completed an average of 32.20 EMA surveys (SD = 11.40) across the 7-day study period. The mean time between completed EMA surveys was 6.64 hours (SD = 2.34), which included overnight gaps and missing responses. The mean response time between completed surveys was partially driven by relatively few data that were collected at very long intervals. In addition, there were 163 surveys that were answered at time intervals below 1 hour, with a mean of 6.52 per participant (see the Supplementary Materials and Methods for a histogram of this data). Therefore, we felt as though rescaling the data to minute intervals was appropriate. The sample size and amount of completed EMA surveys align with the recommended number of timepoints required for estimating continuous-time models [38].

### Drift matrix parameters

**Fig. 2:**
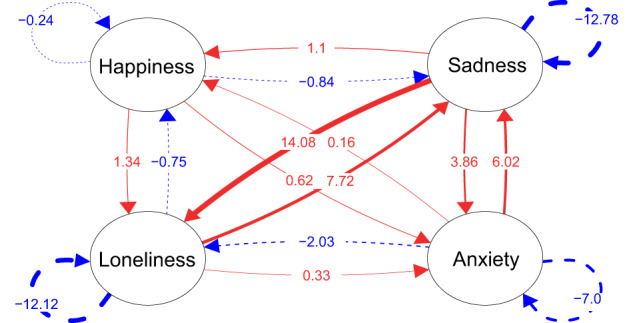
Local dependency network of loneliness and mood states corresponding to the drift matrix parameters. Note. Arrows going into the same variable represent auto-effects and arrows going into different variables represent cross-effects.

The drift matrix parameters are presented in a local dependency network (Fig. 2) and their corresponding ninety-five percent confidence intervals are in Table 1. Red arrows represent positive dependencies, and blue arrows represent negative dependencies. Focusing first on the auto-effects (i.e., circular arrows adjacent to each of the four variables), we see that all values are negative, indicating a stable system. However, there are notable differences among the variables. Specifically, happiness had the lowest auto-effect (−0.24, 95% CI = −0.35, −0.14) by a large margin, indicating that happiness would be expected to return to equilibrium less quickly than loneliness, sadness, and anxiety following a shock to the system. Qualitatively, this means that feelings of loneliness, sadness, and anxiety are more quickly regulated away than happiness, which is more persistent. Notably, loneliness (−12.12, 95%CI = −13.55, −10.71) and sadness (−12.78, 95% CI = −13.80, −11.61) had very similar auto-effects.

**Table 1:** Ninety-five percent Confidence Intervals Corresponding to the Drift Matrix Parameters of Loneliness, Happiness, Sadness, and Anxiety.

	Happiness	Sadness	Anxiety	Loneliness
Happiness	−0.24 (−0.35, −0.14)	1.10 (0.53, 1.67)	0.16 (−0.58, 0.85)	−0.75 (−1.06, −0.45)
Sadness	−0.84 (−1.26, −0.42)	-12.78 (−13.80, −11.61)	6.02 (4.84, 7.17)	7.72 (6.28, 9.07)
Anxiety	0.62 (0.27, 0.97)	3.86 (1.94, 5.62)	−7.00 (−8.57, −5.42)	0.33 (−0.90, 1.60)
Loneliness	1.34 (0.79, 1.82)	14.08 (10.64, 17.33)	−2.03 (−5.46, 1.716)	−12.12 (−13.55, −10.71)

Next, we examined the cross-effects of the local dependency network (Fig. 2). Each variable has a cross-effect to and from every other variable. As a reminder, cross-effects reflect the influence of one variable on another variable’s instantaneous rate of change. The strongest cross-effect was between sadness and loneliness. That is, being sad is likely to lead to a rising trajectory of loneliness. The opposite is also true, such that loneliness is expected to diminish the subsequent rate of change of sadness; however, this effect was weaker in magnitude. Loneliness and happiness showed an unexpected pattern. Loneliness is expected to *decrease* the rate of change of happiness, but happiness *increases* the rate of change of loneliness. This paradoxical relationship can be better understood when we look at the cross-effects of loneliness and happiness over time, which we present in the next section.

### Auto-effects and cross-effects over time

**Fig. 3:**
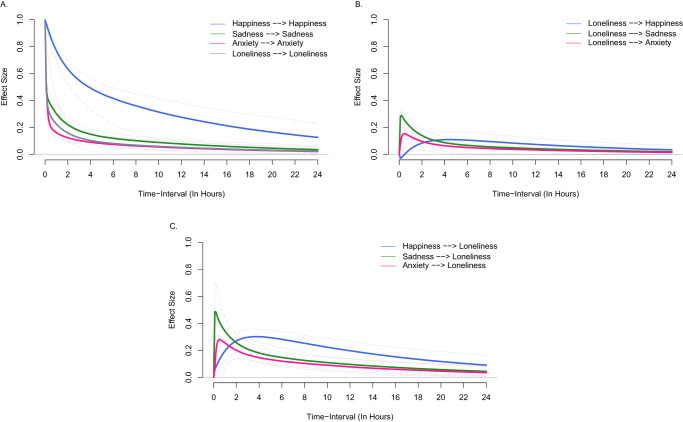
Auto-effects and cross-effects of loneliness and mood states. **A** = Auto-effects; **B** = Cross-effects showing loneliness predicting the rate of change of mood states; **C** = Cross-effects showing mood states predicting the rate of change of loneliness. The dashed lines represent 95% confidence intervals.

To examine how the variables evolve continuously over time, we rescaled the drift matrix parameters to change continuously over 24 hours (Fig. 3, Panel A). Looking at the auto-effects, we see that happiness slowly begins to move back towards equilibrium around two hours later but does not fully reach its equilibrium state within one day. In contrast, loneliness, sadness, and anxiety begin to revert to equilibrium quickly and return to equilibrium after about 20 hours.

For the cross-effects, we examined the influence of momentary loneliness on future mood states (Fig. 3, Panel B) and the influence of momentary mood states on future loneliness (Fig. 3, Panel C). In general, panels B and C show that loneliness and mood states affect each other in slightly different ways. The effect of momentary loneliness on all mood states becomes 0 approximately 19 hours later. However, the effects of momentary mood states on future loneliness do not die down and reach 0 over the course of 24 hours. This result suggests that mood states have a more enduring effect on loneliness than loneliness has on mood states. We can also interpret specific cross-effects. For example, momentary sadness had a stronger effect on the rate of change of future loneliness (0.48, 95% CI = 0.43, 0.54) than momentary loneliness had on the rate of change of future sadness (0.28, 95% CI = 0.24, 0.32), although both effects reach a peak value around 12 minutes (which correspond to the effects and confidence intervals presented above). Meanwhile, the effect of momentary loneliness on the rate of change of future happiness is negative at very short intervals, then becomes positive approximately 42 minutes later; however, this effect does not become significant until the 72-minute mark (0.04, 95% CI = 0.004, 0.08). In contrast, the effect of momentary happiness on the rate of change of future loneliness in panel C slowly builds, reaches its peak around 3 hours later (0.31, 95% CI = 0.25, 0.36), and does not return to 0.

The positive cross-effect of happiness on loneliness may be partly due to a diurnal pattern between the variables. Indeed, we observed that participants tended to have lower levels of loneliness and higher levels of happiness in the morning. Loneliness then tended to rise throughout the day, while happiness tended to lower. We provide more information about this potential diurnal effect in the Supplementary Materials and Methods.

### Impulse response functions

**Fig. 4:**
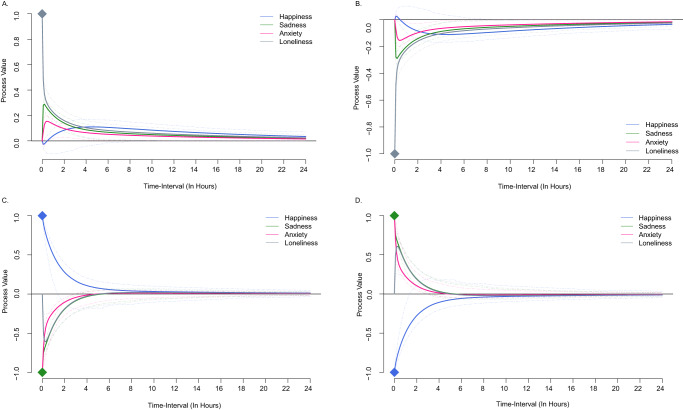
Impulse response functions of loneliness and mood states. Diamonds represent starting positions for impulse responses; Panel **A** = Loneliness with a one standard deviation increase above equilibrium; Panel **B** = Loneliness with a one standard deviation below equilibrium; Panel **C** = Happiness with a one standard deviation above equilibrium and sadness and anxiety with a one standard deviation below equilibrium; Panel **D** = Happiness with a one standard deviation below equilibrium and sadness and anxiety with a one standard deviation above equilibrium. The dashed lines represent 95% confidence intervals.

Impulse response functions are presented in Fig. 4. Panel A shows what would happen to the system if loneliness had a momentary value one standard deviation above equilibrium. When participants feel lonely in the moment, they are expected to experience a decrease in happiness and an increase in sadness and anxiety. Due to its large auto-effect, loneliness begins reverting towards equilibrium instantaneously. Thus, as participants experience less loneliness, they experience less sadness and anxiety and more happiness. Panel B illustrates a negative impulse to loneliness, that is, what we would expect to happen if loneliness took on a value one standard deviation below equilibrium. Here, we can see that as participants experience less loneliness, sadness, and anxiety decrease while happiness temporarily increases. However, as loneliness, sadness, and anxiety approach equilibrium, happiness departs from equilibrium before reverting towards it again after about eight hours.

In panel C, we show an impulse response function corresponding to simultaneous positive impulses in happiness (i.e., more happiness) and negative impulses in sadness and anxiety (i.e., less sadness and anxiety). Loneliness was estimated at the equilibrium value. Participants would be expected to experience decreases in loneliness for about 4 hours until the system quickly reverts to equilibrium. Panel D shows the reverse simultaneous impulses (i.e., less happiness and more sadness and anxiety). As happiness increases towards equilibrium, loneliness, sadness, and anxiety decrease and reach equilibrium around 4 hours later.

## Discussion

Using EMA, researchers can collect ecologically valid data in naturalistic contexts; however, traditional methods used to analyze these data, such as HLM and mlVAR, are limited in their capabilities. These methods model time in discrete measurements even though most psychological processes are thought to change continuously. Continuous-time models, an analytical approach that treats psychological variables as dynamical systems that evolve continuously, overcome these limitations.

To illustrate the interpretive value of continuous-time models, we conducted an empirical demonstration using EMA data of momentary loneliness and mood states in homeless-experienced Veterans with a history of mental illness. The dynamical system was stable, as all variables had negative auto-effects. In addition, we found stronger cross-effects of mood states predicting the rate of change of loneliness over time than vice versa, with the strongest effect emerging between momentary sadness and the rate of change of loneliness 12 minutes later. This finding could be used to inform future EMA studies that aim to examine the relationship between loneliness and mood. Researchers may want to use a very short sampling interval or use other sampling strategies, such as a burst design, to capture peak effect sizes between the variables. Regarding impulse response functions, we found that the rate of change among loneliness, sadness, and anxiety decreases in response to a sudden spike in loneliness. In contrast, the rate of change of happiness temporarily increases over time in response to a sudden spike in loneliness. Overall, this demonstration highlights the ability of continuous-time models to answer substantively interesting and clinically relevant questions related to auto-effects, cross-effects, and impulse response predictions.

One potential limitation of our demonstration relates to the equilibrium value. For ease of interpretation, all variables had the same global equilibrium value of 0, a common practice within psychological literature. However, it is possible that some variables had multiple equilibria or that the equilibrium of certain variables depended on the values of other variables [40]. If some equilibrium depended on the values of other variables, the dynamical system would be better conceptualized as nonlinear. In contrast to a linear system in which the equilibrium is stable over time, the equilibrium within a nonlinear system may exhibit oscillations over time, such as sinusoid functions. More complex continuous-time models will need to be tested to truly understand the temporal dynamics of psychological variables. Another potential limitation was that we rescaled the drift matrix parameters to minute-to-minute intervals despite the average time interval between measurements being approximately 6 hours per participant. Although our theoretical understanding suggests that loneliness and mood states can fluctuate at minute intervals, along with having a moderate amount of actual data collected at time intervals under 1 hour, the results for effects at minute-to-minute intervals may reflect some biases. That said, researchers should use caution when interpolating or extrapolating findings from continuous-time models beyond a reasonable time interval.

Besides applying continuous-time models to EMA or other forms of conventual longitudinal data, these models could also be used to analyze different types of neuroimaging data, such as electroencephalography (EEG) and functional magnetic resonance imaging (fMRI). Applying continuous-time models to EEG and fMRI data would allow for a nuanced understanding of brain activity and the ability to precisely capture complex, rapidly changing patterns. This approach could be valuable for neuroscience research and clinical diagnostics by offering enhanced insights into brain function and the potential for more accurate detection of neurological conditions. An exciting avenue for extending the clinical application of continuous-time models is using network centrality metrics to identify intervention targets [5, 40]. Network centrality metrics for continuous-time models can provide information on the relative importance of individual variables and which variables would be optimal intervention targets. Using this information, we could design an intervention that aims to change a variable’s value over time, also known as a “press” intervention [5, 40]. For example, we may want to induce a change in sadness levels over 2 hours by prompting participants to view positively valanced stimuli (like a picture or video), pre-prepared positive affirmation statements, or other standard coping techniques from cognitive behavioral-based interventions. Once the intervention holds sadness levels constant, we can observe how loneliness, happiness, and anxiety change over time. This example is one of the many ways researchers could leverage continuous-time models to construct clinically relevant interventions.

### Citation diversity statement

The authors have attested that they made efforts to be mindful of diversity in selecting the citations used in this article.

## Supplementary information

Supplementary Materials and Methods for Applying continuous-time models to ecological momentary assessments: A practical introduction to the method and demonstration with clinical data

## References

[1] Shiffman S, Stone AA, Hufford MR. Ecological momentary assessment. Annu Rev Clin Psychol. 2008;4:1–32. 18509902 10.1146/annurev.clinpsy.3.022806.091415

[2] Voelkle MC, Oud JH, Davidov E, Schmidt P. An SEM approach to continuous time modeling of panel data: relating authoritarianism and anomia. Psychol Methods. 2012;17:176–92. 22486576 10.1037/a0027543

[3] de Haan-Rietdijk S, Voelkle MC, Keijsers L, Hamaker EL. Discrete- vs. continuous-time modeling of unequally spaced experience sampling method data. Front Psychol. 2017;8:1849. 29104554 10.3389/fpsyg.2017.01849 PMC5655034

[4] Voelkle MC, Oud JH. Continuous time modelling with individually varying time intervals for oscillating and non-oscillating processes. Br J Math Stat Psychol. 2013;66:103–26. 22420323 10.1111/j.2044-8317.2012.02043.x

[5] Ryan O, Hamaker EL. Time to Intervene: A continuous-time approach to network analysis and centrality. Psychometrika. 2022;87:214–52. 34165691 10.1007/s11336-021-09767-0 PMC9021117

[6] Ryan O, Kuiper RM, Hamaker EL: A continuous-time approach to intensive longitudinal data: what, why and how? In: Continuous time modeling in the behavioral and related sciences, editors Montfort KV, Oud JHL, Voelkle MC. New York, NY: Springer; 2018. 27–54.

[7] Stone AA, Schneider S, Smyth JM. Evaluation of pressing issues in ecological momentary assessment. Annu Rev Clin Psychol. 2023;19:107–31. 36475718 10.1146/annurev-clinpsy-080921-083128 PMC12991416

[8] Woltman H, Feldstain A, MacKay JC, Rocchi M. An introduction to hierarchical linear modeling. Tutorials Quant Methods Psychology. 2012;8:52–69.

[9] Bringmann LF, Vissers N, Wichers M, Geschwind N, Kuppens P, Peeters F, et al. A network approach to psychopathology: new insights into clinical longitudinal data. PLoS One. 2013;8:e60188. 23593171 10.1371/journal.pone.0060188 PMC3617177

[10] Hamaker EL, Kuiper RM, Grasman RP. A critique of the cross-lagged panel model. Psychol Methods. 2015;20:102–16. 25822208 10.1037/a0038889

[11] Epskamp S, Waldorp LJ, Mõttus R, Borsboom D. The Gaussian Graphical Model in cross-sectional and time-series data. Multivariate Behav Res. 2018;53:453–80. 29658809 10.1080/00273171.2018.1454823

[12] Hamaker EL, Wichers M. No time like the present: discovering the hidden dynamics in intensive longitudinal data. Curr Dir Psychol Sci. 2017;26:10–15.

[13] Batra R, Johal SK, Chen M, Ferrer E. Consequences of sampling frequency on the estimated dynamics of AR processes using continuous-time models. *Psychol Methods*. Published online July 10, 2023. 10.1037/met0000595 37428727

[14] Mülken O, Blumen A. Continuous-time quantum walks: models for coherent transport on complex networks. Phys. Rep. 2011;502:37–87.

[15] Andersen TG, Benzoni L, Lund J. An empirical investigation of continuous-time equity return models. J. Finance. 2002;57:1239–84.

[16] Andersen TG, Bollerslev T, Frederiksen P, Ørregaard Nielsen M. Continuous-time models, realized volatilities, and testable distributional implications for daily stock returns. J. Appl. Econ. 2010;25:233–61.

[17] Coppersmith DDL, Ryan O, Fortgang RG, Millner AJ, Kleiman EM, Nock MK. Mapping the timescale of suicidal thinking. Proc Natl Acad Sci USA. 2023;120:e2215434120. 37071683 10.1073/pnas.2215434120 PMC10151607

[18] Moggia D, Bennemann B, Schwartz B, Hehlmann MI, Driver CC, Lutz W. Process-Based psychotherapy personalization: considering causality with continuous-time dynamic modeling. Psychother Res. 2023;33:1076–95. 37306112 10.1080/10503307.2023.2222892

[19] Ruissen GR, Beauchamp MR, Puterman E, Zumbo BD, Rhodes RE, Hives BA, et al. Continuous-time modeling of the bidirectional relationship between incidental affect and physical activity. Ann Behav Med. 2022;56:1284–99. 35802004 10.1093/abm/kaac024 PMC9672348

[20] Hauger GS. Instantaneous rate of change: a numerical approach. Int J Math Educ Sci Technol. 2000;31:891–7.

[21] Särkkä S, Solin A. *Applied Stochastic Differential Equations*. Cambridge University Press. 2019. 324 p. (Institute of Mathematical Statistics Textbooks).

[22] Sobczyk K. *Stochastic differential equations: with applications to physics and engineering* (Vol. 40). 2001. Springer Science & Business Media.

[23] Driver CC, Voelkle MC. Hierarchical Bayesian continuous time dynamic modeling. Psychol Methods. 2018;23:774–99. 29595295 10.1037/met0000168

[24] Hamilton JD. *Time series analysis* (Vol. 2). 1994. Princeton: Princeton University Press.

[25] Oravecz Z, Tuerlinckx F, Vandekerckhove J. A hierarchical latent stochastic differential equation model for affective dynamics. Psychol. Methods. 2011;16:468–90. 21823796 10.1037/a0024375

[26] Adolf JK, Loossens T, Tuerlinckx F, Ceulemans E. Optimal sampling rates for reliable continuous-time first-order autoregressive and vector autoregressive modeling. Psychol Methods. 2021;26:701–18. 34166049 10.1037/met0000398

[27] Driver CC, Voelkle, MC Understanding the time course of interventions with continuous time dynamic models. In KL Montfort, JH Oud, MC Voelkle (Eds.). *Continuous time modeling in the behavioral and related sciences*. New York: Springer;2018. 179–203.

[28] Hawkley LC, Cacioppo JT. Loneliness matters: a theoretical and empirical review of consequences and mechanisms. Ann Behav Med. 2010;40:218–27. 20652462 10.1007/s12160-010-9210-8 PMC3874845

[29] Valtorta N, Hanratty B. Loneliness, isolation and the health of older adults: do we need a new research agenda? J R Soc Med. 2012;105:518–22. 23288086 10.1258/jrsm.2012.120128 PMC3536512

[30] Cacioppo JT, Cacioppo S, Boomsma DI. Evolutionary mechanisms for loneliness. Cogn Emot. 2014;28:3–21. 24067110 10.1080/02699931.2013.837379 PMC3855545

[31] Matthews GA, Tye KM. Neural mechanisms of social homeostasis. Ann N Y Acad Sci. 2019;1457:5–25. 30875095 10.1111/nyas.14016 PMC7593988

[32] Cacioppo JT, Hawkley LC, Ernst J, Burleson M, Berntson G, Nouriani B, et al. Loneliness within a nomological net: An evolutionary perspective. J. Res. Pers. 2006;40:1054–85.

[33] Dahlberg L, McKee KJ, Frank A, Naseer M. A systematic review of longitudinal risk factors for loneliness in older adults. Aging Ment Health. 2022;26:225–49. 33563024 10.1080/13607863.2021.1876638

[34] van Roekel E, Verhagen M, Engels RCME, Scholte RHJ, Cacioppo S, Cacioppo JT. Trait and state levels of loneliness in early and late adolescents: examining the differential reactivity hypothesis. J Clin Child Adolesc Psychol. 2018;47:888–99. 27191708 10.1080/15374416.2016.1146993

[35] Buecker S, Horstmann KT, Luhmann M. Lonely today, lonely tomorrow: Temporal dynamics of loneliness in everyday life and its associations with psychopathological symptoms. Soc. Psychol. Pers. Sci. 2024;15:170–181.

[36] Fisher AJ, Reeves JW, Lawyer G, Medaglia JD, Rubel JA. Exploring the idiographic dynamics of mood and anxiety via network analysis. J Abnorm Psychol. 2017;126:1044–56. 29154565 10.1037/abn0000311

[37] First MB, Williams JBW, Karg RS, Spitzer RL*:**Structured Clinical Interview for DSM-5 Disorders, Clinician Version (SCID-5-CV)*. Arlington, VA, American Psychiatric Association, 2016.

[38] Hecht M, Zitzmann S. Sample size recommendations for continuous-time models: Compensating shorter time series with larger numbers of persons and vice versa. Struct. Equ. Model. 2021;28:229–36.

[39] Driver CC, Oud JHL, Voelkle MC. Continuous time structural equation modeling with R Package ctsem. J. Stat. Soft. 2017;77:1–35.

[40] Ryan O, Dablander F. Equilibrium Causal Models: ConnEcting Dynamical Systems Modeling And Cross-sectional Data Analysis. https://psyarxiv.com/q4d9g. 2022. 10.1080/00273171.2025.2522733 40904318

