# Supplementary material for: Applying continuous-time models to ecological momentary assessments: A practical introduction to the method and demonstration with clinical data
Source: NPP Digit Psychiatry Neurosci. 2024 Mar 7;2:2. doi: 10.1038/s44277-024-00004-x (PMC12624882; doi:10.1038/s44277-024-00004-x)
Supplement: Supplementary file 1 — Supplementary Materials and Methods for Applying continuous-time models to ecological momentary assessments: A practical introduction to the method and demonstration with clinical data [file 44277_2024_4_MOESM1_ESM.docx]

**Supplementary Materials and Methods for Applying continuous-time models to ecological momentary assessments:**

**A practical introduction to the method and demonstration with clinical data**

**Supplementary Information 1:** Distribution of Time in Between Completed EMA Surveys.

**Supplementary Information 2 :** Diurnal Effect Between Loneliness and Happiness.

**Supplementary Figure 1 :** Histogram of Time in Between Completed EMA Surveys Across All Participants.

**Supplementary Figure 2:** Average Levels of Loneliness and Happiness Throughout the Day.

**Supplementary Information 1:** The mean time in between survey responses was 6.64 hours; however, this value was partially driven by surveys completed between very long time intervals (e.g., 40 hours). We present a histogram in Supplementary Figure 1 that shows the distribution of time in between completed surveys across the sample.

**Supplementary Information 2:** We observed an unexpected cross-lagged effect of happiness on loneliness. Specifically, we observed that happiness in the moment predicts future loneliness, with the peak of this effect occurring approximately 4 hours later. While this effect may seem contradictory to expectation, it could stem from diurnal variation among the variables. Diurnal variation is often associated with depression, such that people with depression may experience lower mood and higher levels of depressive symptoms in the morning. The severity of these symptoms can weaken, and mood thus improves as the day progresses.

Given that over half of our sample had a depressive disorder diagnosis, it is possible that our continuous time models were capturing a diurnal effect between happiness and loneliness. Supplementary Figure 1 illustrates this possibility, as it shows that participants had low levels of loneliness and high levels of happiness in the morning; loneliness gradually increased, and happiness gradually decreased. Another potential explanation is that happiness decreased, and loneliness increased due to a third variable, such as the quality and quantity of social interactions.

**
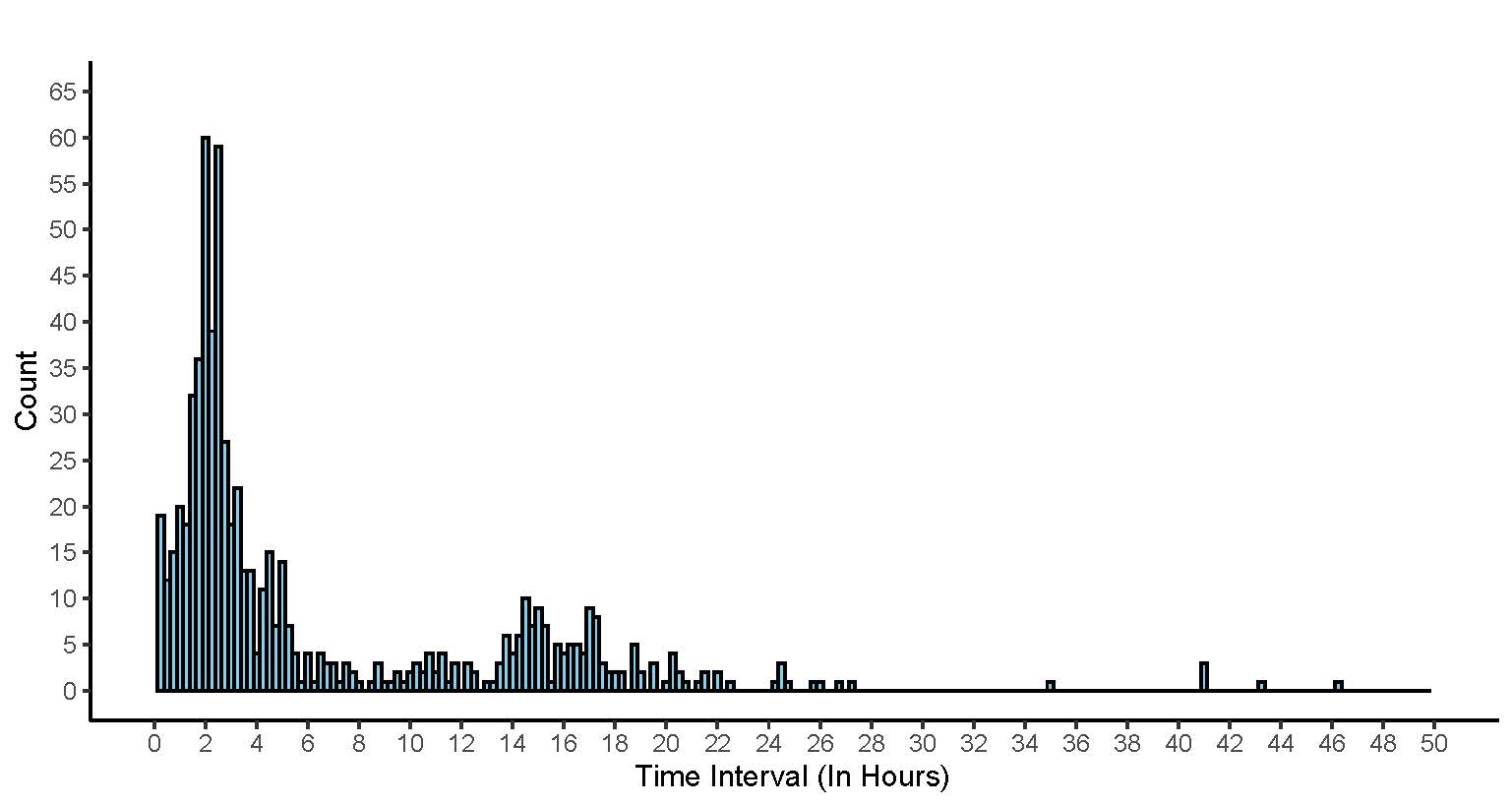
**

**Supplementary Figure 1.** Histogram of Time in Between Completed EMA Surveys Across All Participants.


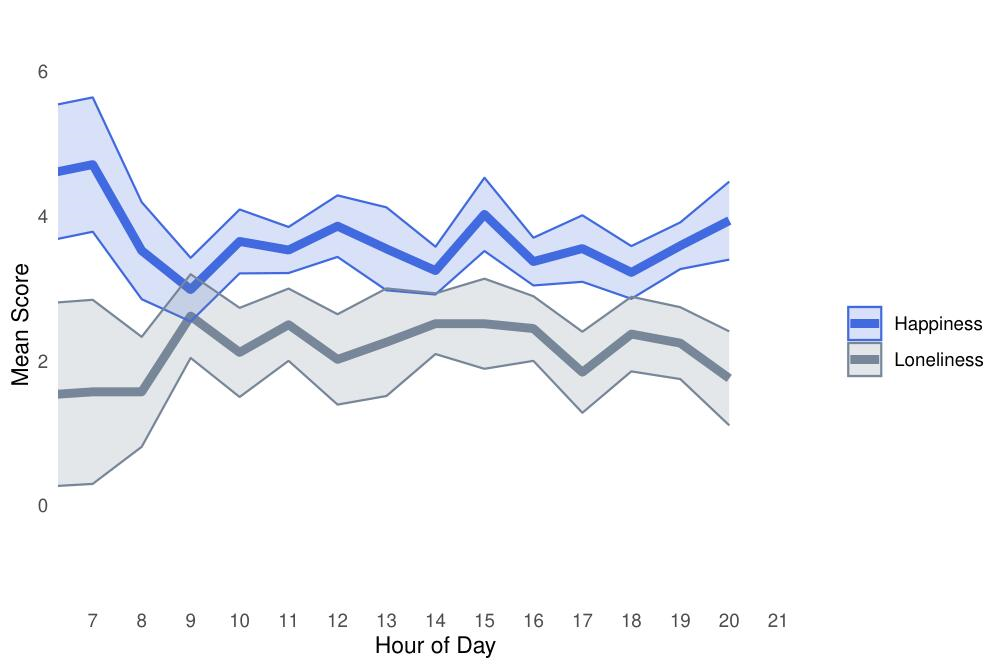


**Supplementary Figure 2.** Average Levels of Loneliness and Happiness Throughout the Day.
